# Effect of Surface Anions Adsorbed by Rutile TiO_2_ (001) on Photocatalytic Nitrogen Reduction Reaction: A Density Functional Theory Calculation

**DOI:** 10.3390/molecules29194566

**Published:** 2024-09-25

**Authors:** Xiaoyu Jiang, Mengyuan Gao, Hongda Li

**Affiliations:** 1 Suzhou Institute for Advanced Research, University of Science and Technology of China, Suzhou 215123, China; 2 School of Materials Science and Engineering, Henan Normal University, Xinxiang 453007, China; 3 School of Chemistry and Chemical Engineering, Hubei Normal University, Huangshi 435002, China

**Keywords:** rutile TiO_2_, nitrogen reduction reaction (NRR), DFT calculation, anion adsorption, photocatalysis

## Abstract

The adsorption of common anions found in water can have a considerable impact on the surface state and optical characteristics of titanium dioxide (TiO_2_), which has an important impact on the photocatalytic nitrogen reduction reaction (NRR). This work utilizes density functional theory (DFT) computations to examine the electronic and optical characteristics of the TiO_2_ (001) surface under various anion adsorptions in order to clarify their influence on the photocatalytic NRR of TiO_2_. The modifications in the structure, optical, and electronic properties of TiO_2_ before and after anion adsorption are investigated. In addition, the routes of Gibbs free energy for the NRR are also evaluated. The results indicate that the adsorption of anions modifies the surface characteristics of TiO_2_ to a certain degree, hence impacting the separating and recombining charge carriers by affecting the energy gap of TiO_2_. More importantly, the adsorption of anions can increase the energy barriers for the NRR, thereby exerting a detrimental effect on its photocatalytic activity. These findings provide a valuable theoretical contribution to understanding the photocatalytic reaction process of TiO_2_ and its potential application of NRR in the actual complex water phase.

## 1. Introduction

Due to constant population growth and the increasing tempo of industrialization, the problems concerned with energy and environmental deterioration have become more severe. Conventional resources of energy are scarce, and many of them are dwindling in their capacity to meet the growing demand [1]. Therefore, the challenge of constant energy consumption has led to research and development on renewable, clean, and efficient energy technologies. Photocatalysis, as one of the key approaches, has shown great promise in addressing energy limitations [2,3].

Of all the photocatalytic materials, TiO_2_ stands out due to its favorable electronic structure; it can generate photo-induced electron–hole pairs under sunlight [4,5]. Due to its high chemical resistance and cheap price, it has become the object of research focus in the photocatalysis area [6]. Commercially, TiO_2_ has been widely used in various photocatalytic reactions, includes hydrogen/oxygen production by water splitting [7,8], degradation of organic pollutants [9,10], CO_2_ reduction [11], nitrogen reduction reaction (NRR) for ammonia synthesis, and so on [12]. Among them, the eco-friendly photocatalytic NRR is particularly prominent; it can convert N_2_ into NH_3_ at normal temperature and pressure, and the product, NH_3,_ is one of the key chemicals for maintaining the continuation of life on earth and meeting the needs of human society in the energy and chemical industries.

Rutile and anatase are two types of crystal phases of TiO_2_ recognized for their superior electrical properties and photocatalytic efficiency [13]. Rutile TiO_2_ is renowned for its exceptional chemical stability, particularly in demanding industrial settings characterized by high temperatures and severe pH levels [14]. Rutile TiO_2_ has a wider bandgap; the material has a high photocatalytic activity mainly under UV light, and hence is ideal for photocatalytic processes that require effective separation of photogenerated electron–hole pairs [15]. A natural TiO_2_ crystal predominantly exposes the (110), (111), and (001) facets. Among them, the (001) facet exhibits significantly higher normalized surface photo reactivity [16].

Photocatalytic processes with TiO_2_ as the catalyst begin with the absorption of light. TiO_2_ effectively produces electron–hole pairs when exposed to UV light. These pairs can then engage in redox processes [17]. The reactions mostly take place on the surface of TiO_2_, where the reactants are adsorbed and interact. The surface characteristics of TiO_2_ play a crucial role in determining its total photocatalytic effectiveness [18,19]. Surface adsorption capacity, defect states, and surface charge distribution have a direct impact on the activation of reactants and the routes of reactions [20].

TiO_2_ is extensively employed in aqueous photocatalytic reactions [21]. Nevertheless, real water environments consist of a multitude of anions, including fluoride (F^−^), chloride (Cl^−^), bromide (Br^−^), iodide (I^−^), sulfate (SO_4_^2−^), nitrate (NO_3_^−^), and carbonate (CO_3_^2−^) [22]. These anions readily adsorb onto the TiO_2_ surface, potentially introducing surface states, altering the positions of the valence and conduction bands, and thereby affecting the bandgap. This in turn impacts the photocatalytic performance of TiO_2_ [23]. Therefore, it is of great practical interest to examine the adsorption effects of these anions on the surface of TiO_2_ and their impact on photocatalytic performance.

We conducted a comprehensive study on how the adsorption of common aqueous anions, including F^−^, Cl^−^, Br^−^, I^−^, NO_3_^−^, CO_3_^2−^, and SO_4_^2−^, affects the electrical and optical characteristics of the TiO_2_ (001) surface. Using density functional theory (DFT) calculations, we analyzed changes in the bandgap, density of states (DOSs), optical absorption spectra, and Mulliken charge distribution before and after anion adsorption. Moreover, we examined the effects of anion adsorption on the Gibbs free energy of the NRR on TiO_2_. This proves that anion adsorption does indeed affect the photocatalytic behavior of TiO_2_ in a negative manner. These findings not only provide a significant theoretical framework and guidance for using TiO_2_ photocatalysts in multicomponent aqueous systems, but are also applicable to electrocatalytic nitrogen fixation, further expanding its potential applications in clean energy technologies [24,25].

## 2. Results and Discussion

### 2.1. Adsorption Models and Adsorption Energies

**Figure 1:**
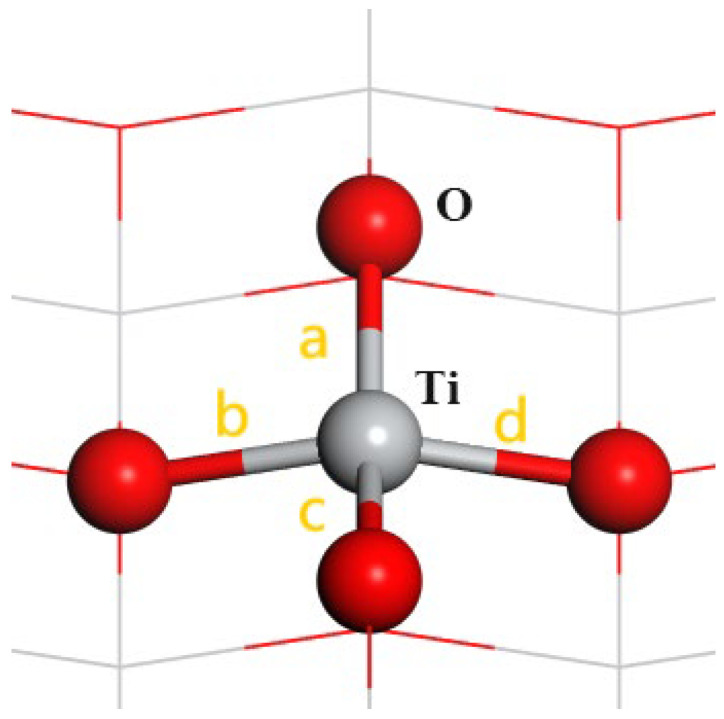
Model of the rutile TiO_2_ (001) surface. The four Ti-O bonds connected by Ti atoms below the adsorbed ion are a, b, c and d; Gray ball is Ti atom, red balls are O atom.

**Figure 2:**
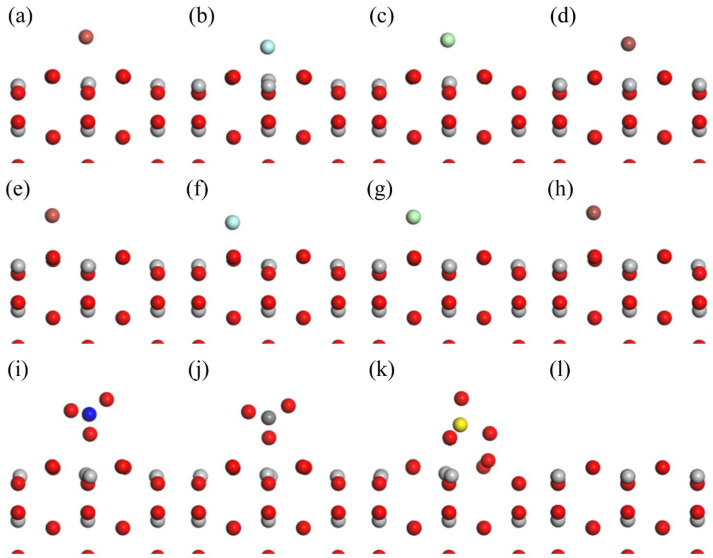
The models before and after anion adsorption: (**a**–**l**) represent TiO_2_-Ti-F^−^, TiO_2_-Ti-Cl^−^, TiO_2_-Ti-Br^−^, TiO_2_-Ti-I^−^, TiO_2_-O-F^−^, TiO_2_-O-Cl^−^, TiO_2_-O-Br^−^, TiO_2_-O-I^−^, TiO_2_-NO_3_^−^, TiO_2_-CO_3_^2−^, TiO_2_-SO_4_^2−^, and TiO_2_, respectively.

As shown in Figure 1 and Figure 2, the study focuses on the adsorption of halide anions on two specific spots of the rutile TiO_2_ (001) surface, namely, directly above the O atom and directly above the Ti atom. These sites are denoted as TiO_2_-O-X^−^ and TiO_2_-Ti-X^−^ (X = F, Cl, Br, I), respectively. For the adsorption of NO_3_^−^, CO_3_^2−,^ and SO_4_^2−^ anions, only the adsorption site directly above the Ti atom was considered, denoted as TiO_2_-NO_3_^−^, TiO_2_-CO_3_^2−^, and TiO_2_-SO_4_^2−^, respectively.

Table 1 provides a thorough examination of the changes in bond length and adsorption energy on the rutile TiO_2_ (001) surface when different anions are adsorbed. We labeled the four edges surrounding the Ti atom beneath the adsorbed ion as a, b, c, and d, and measured the average lengths of these edges for the eight Ti-O bonds directly below. The results show that the adsorption of all anions causes an elongation of these O-Ti bonds. Among the halide anions adsorbed at the Ti site (F^−^, Cl^−^, Br^−^, I^−^), F^−^ induces the most significant bond elongation, with the bond length reaching 1.950 Å. This is complemented by the most negative adsorption energy of −3.505 eV. As the atomic radius of the halogen increases from Cl^−^ to I^−^, both the adsorption energy and the extent of bond elongation decrease accordingly. When comparing the adsorption of halide anions at the Ti and O sites, it is discovered that adsorption at the O site leads to a somewhat lower elongation of the O-Ti bond compared to the Ti site. Likewise, the adsorption energies at the O site regularly exhibit less negative values compared to those at the Ti site. Therefore, all the following computations were conducted with the Ti site.

Besides halide anions, the study of complex anion adsorption (NO_3_^−^, CO_3_^2−^, SO_4_^2−^) on the surface structure of TiO_2_ is also examined in this work. It is found that all three anions lead to good chemisorption, with the SO_4_^2−^ anion having the highest effect because of its large multi-atomic anion. This includes an adsorption energy of −3.025 eV and the largest degree of bond extension by 1.950 Å. These facts show that the interaction of complex anions in the solution with the TiO_2_ surface may lead to impressive distortions of the crystal lattice.

### 2.2. Electronic Structure

**Figure 3:**
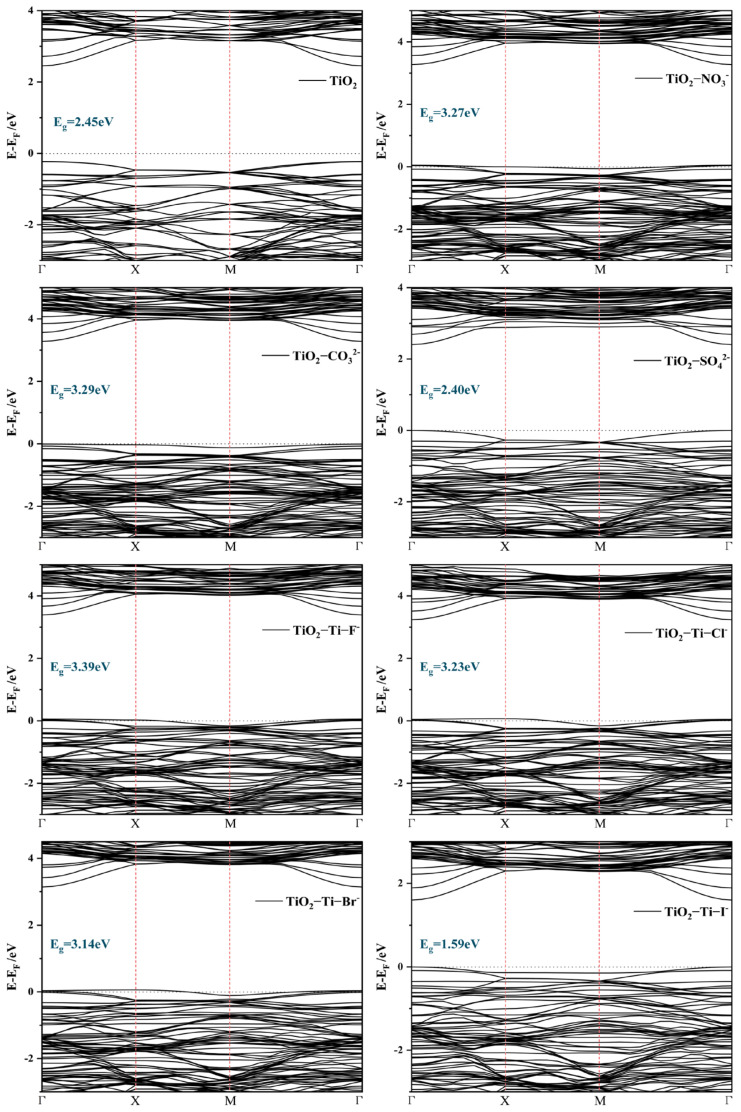
The band structures of TiO_2_, TiO_2_-NO_3_^−^, TiO_2_-CO_3_^2−^, TiO_2_-SO_4_^2−^, TiO_2_-Ti-F^−^, TiO_2_-Ti-Cl^−^, TiO_2_-Ti-Br^−^, and TiO_2_-Ti-I^−^. The red dotted line represents high symmetry points.

**Figure 4:**
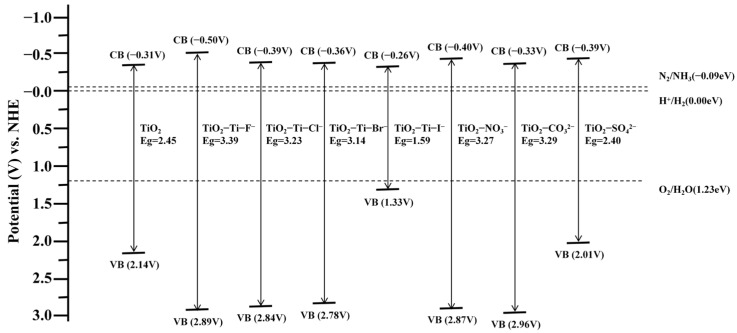
The band alignments before and after anion adsorption.

The changes in the band structure are of critical importance to the photocatalytic activity of TiO_2_ as they determine the material’s optical response range and electron transport characteristics [26]. In general, a narrower bandgap enables the material to capture a wider variety of light wavelengths, which in turn increases its ability to catalyze chemical reactions when exposed to visible light [27]. Nonetheless, a desirable bandgap is when it is not too small since it may lead to early recombination of electron–hole pairs, thus reducing the photocatalysis effect [28]. In this study, we calculated the interfacial bandgap and band alignments of TiO_2_ before and after anion adsorption, as illustrated in Figure 3 and Figure 4, respectively. The pristine rutile TiO_2_ (001) surface exhibits a bandgap of 2.45 eV. Significant alterations in the bandgap were detected with the adsorption of anions. For halide anions adsorbed at the Ti site, the bandgaps of the TiO_2_-Ti-F^−^, TiO_2_-Ti-Cl^−^, and TiO_2_-Ti-Br^−^ systems increased to 3.39 eV, 3.23 eV, and 3.14 eV, respectively, indicating a widening of the bandgap, with fluorine having the most pronounced effect. In contrast, the TiO_2_-Ti-I^−^ system, following I^−^ adsorption, likely formed localized states, leading to a substantial reduction in the bandgap to 1.59 eV.

The bandgaps of the TiO_2_-NO_3_^−^, TiO_2_-CO_3_^2−^, and TiO_2_-SO_4_^2−^ systems were calculated to be 3.27 eV, 3.29 eV, and 2.40 eV, respectively, for the adsorption of complex anions. The substantial increase in bandgap resulting from the adsorption of NO_3_^−^ and CO_3_^2−^ indicates a robust electrical interaction with the surface of TiO_2_. In contrast, the little reduction in the bandgap after the adsorption of SO_4_^2−^ might be ascribed to the bulky molecular structure of the sulfate ion, which could lead to electronic rearrangement on the surface.

**Figure 5:**
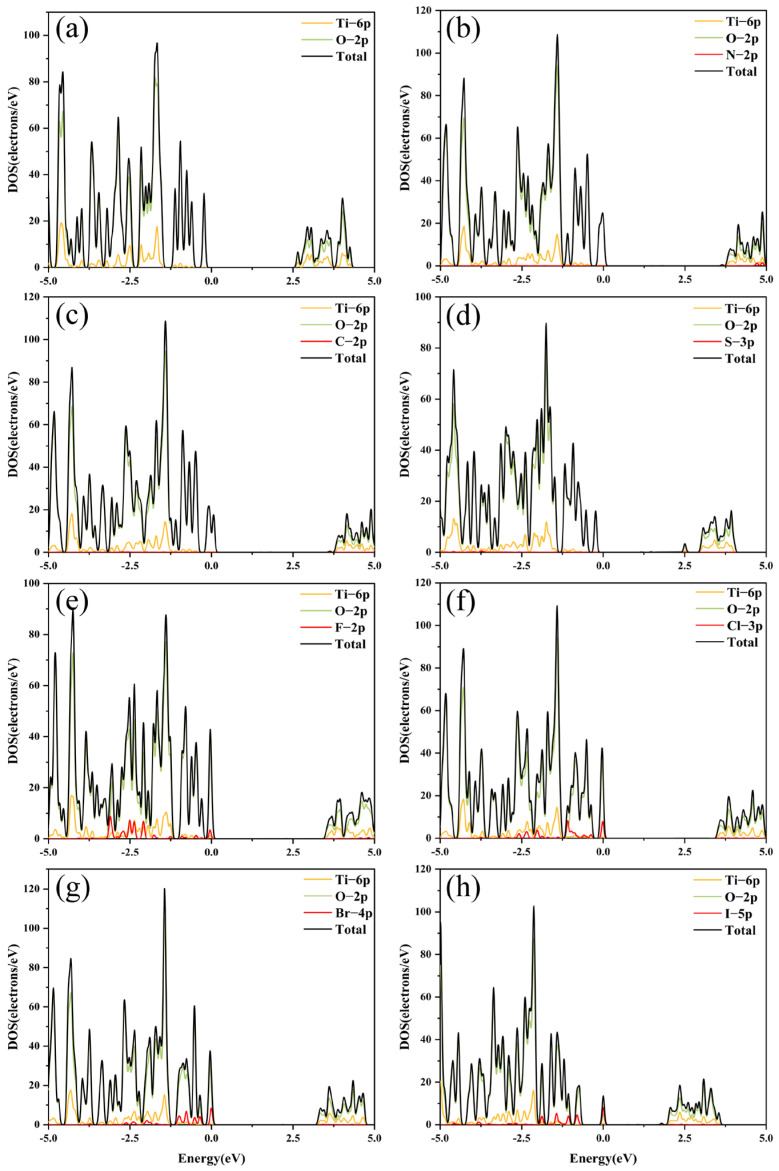
(**a**–**h**) Density of states of TiO_2_, TiO_2_-NO_3_^−^, TiO_2_-CO_3_^2−^, TiO_2_-SO_4_^2−^, TiO_2_-Ti-F^−^, TiO_2_-Ti-Cl^−^, TiO_2_-Ti-Br^−^, and TiO_2_-Ti-I^−^.

The density of states (DOSs) analysis is a powerful tool for elucidating the distribution of electrons across various energy levels and for identifying the contributions of specific atoms and orbitals to the overall electronic structure. In Figure 5, the DOSs of the TiO_2_ show that the valence band (VB) is mainly composed of the oxygen 2p orbitals, while the conduction band (CB) is mainly composed of titanium 3D orbitals. The analysis of the electronic structure of TiO_2_ revealed that the adsorption of NO_3_^−^, CO_3_^2−^, and SO_4_^2−^ organic anions has a weak impact on the material. This suggests that the N, C, and S orbitals of these complex anions have a negligible impact on the valence and conduction bands of TiO_2_.

Nevertheless, a noticeable change in the electronic configuration is noticed when halogen anions are absorbed into the surface of TiO_2_. The outer orbitals of the halogen atoms not only contribute to the modification of the valence band but also lead to the formation of localized states, particularly in the case of I^−^. The emergence of these localized states around the Fermi level leads to a substantial decrease in the band gap of TiO_2_. In contrast, the adsorption of F^−^ and Cl^−^ induces a relatively broad energy dispersion, whereas Br^−^ exhibits the most pronounced energy localization. This localization may give rise to deep-level defect states, potentially impacting the photoelectric properties of the material.

The Mulliken charge analysis data, as shown in Table 2, provide a detailed view of the electron transfer dynamics within the TiO_2_ (001) surface following the adsorption of various complex anions. Among the complex anions studied, the TiO_2_-NO_3_^−^ complex exhibits the most pronounced electron transfer, indicating a strong interaction between the NO_3_^−^ anion and the TiO_2_ surface. This is followed by TiO_2_-CO_3_^2−^ and TiO_2_-SO_4_^2−^, both of which also show significant, albeit varying, degrees of electron transfer.

In systems involving halogen anion adsorption, the TiO_2_-Ti-I^−^ complex demonstrates the highest level of electron transfer, followed by TiO_2_-Ti-F^−^, TiO_2_-Ti-Cl^−^, and TiO_2_-Ti-Br^−^. The adsorption of all these anions results in a redistribution of electrons across the TiO_2_ surface.

After adsorption, the electron density is mostly concentrated around the adsorbed anions, resulting in a decrease in electron density on the TiO_2_ (001) surface. This redistribution of electrons alters the electronic environment of the TiO_2_ surface, thereby modifying its surface properties and potentially influencing its catalytic behavior.

### 2.3. Optical Absorption

The optical absorption spectrum can be obtained through linear response theory. First, the real and imaginary parts of the dielectric function (ε(ω)) are calculated, and then the optical absorption spectrum is derived by analyzing the imaginary part of the dielectric function (Im ε(ω)).

**Figure 6:**
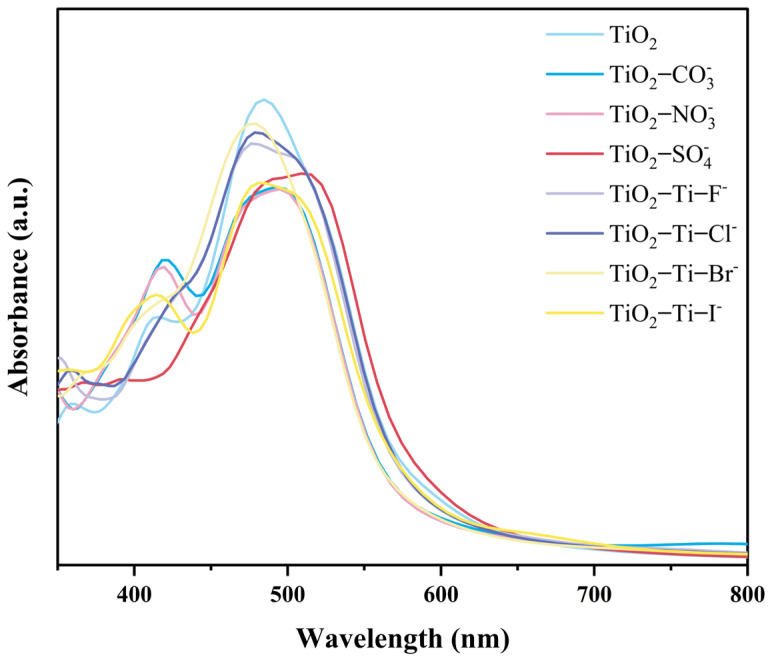
The optical absorption spectra before and after anion adsorption.

The UV-Vis absorption spectra of TiO_2_ samples before and after anion adsorption were measured, as depicted in Figure 6. It is evident that anion adsorption significantly alters the optical properties of TiO_2_. The pristine TiO_2_ sample exhibits a strong absorption peak around 480 nm. However, following anion adsorption, the samples display varying degrees of peak shifts and changes in absorption intensity.

For TiO_2_ adsorbing NO_3_^−^, CO_3_^2−^, and SO_4_^2−^, while the primary absorption characteristics remain similar to those of pristine TiO_2_, the intensity changes are more pronounced, particularly with a noticeable decrease in the absorption intensity near 480 nm. On the other hand, the adsorption of halide anions (F^−^, Cl^−^, Br^−^, and I^−^) leads to significant red shifts and blue shifts in the TiO_2_ absorption peaks. The absorption intensity is notably higher compared to the samples adsorbing NO_3_^−^, CO_3_^2−^, and SO_4_^2−^. Specifically, the adsorption of F^−^ and Cl^−^ results in a marked red-shift of the TiO_2_ absorption peak, extending the absorption range to longer wavelengths. In comparison, the adsorption of Br^–^ and I^−^ causes smaller peak shifts but still affects the absorption characteristics.

Overall, the adsorption of anions induces substantial modifications in the optical absorption properties of TiO_2_, particularly affecting its visible light absorption characteristics.

### 2.4. NRR Gibbs Free Energy Pathway

The nitrogen reduction reaction (NRR) shows great potential in the fields of environmental protection and energy production, particularly in the realms of sustainable agriculture and green chemistry [29]. The synthesis of ammonia (NH_3_) is a critical process in modern industry, serving as a key intermediate in fertilizer production and, potentially, as a hydrogen carrier in the future, offering a clean and efficient energy solution. The nitrogen molecule (N_2_) is characterized by an exceptionally high bond dissociation energy (approximately 941 kJ/mol) and the extraordinary stability of the N≡N triple bond [30]. Therefore, a catalyst capable of effectively activating and reducing N_2_ must possess robust electron transfer capabilities and stable reactive sites. These sites need to effectively adsorb and activate N_2_ molecules, as well as support the consecutive electron transfer and hydrogenation processes necessary for the reduction of N_2_ to NH_3_. Therefore, it is necessary to have catalyst materials with outstanding surface electronic properties that can be consistently maintained to facilitate uninterrupted photocatalytic reactions. TiO_2_ with its efficient charge separation capabilities and chemical stability, has emerged as a focal point in photocatalytic NRR research [31]. Anions present in water include F^−^, Cl^−^, Br^−^, I^−^, NO_3_^−^, CO_3_^2−^, and SO_4_^2−^ adsorb on the surface of TiO_2_ and modify the electronic structure, and therefore, the activity of the catalyst in NRR. Consequently, among the various photocatalytic reactions, we have prioritized NRR for evaluating catalyst efficiency. Investigating the catalytic performance of TiO_2_ before and after anion adsorption will be useful for the improvement of TiO_2_-based NRR catalysts.

**Figure 7:**
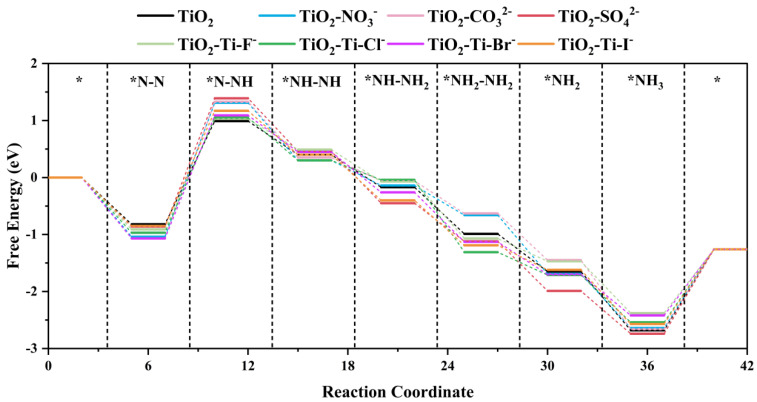
The Gibbs free energy for NRR on TiO_2_ before and after anion adsorption. “*” represents free electrons.

The photocatalytic NRR commonly follows the alternating hydrogenation mechanism as the predominant reaction pathway [32,33]. In order to investigate the impact of anion adsorption on the photocatalytic performance of TiO_2_, we examined the changes in Gibbs free energy for all materials involved in this process, as depicted in Figure 7. The initial hydrogenation step (Equation (2)) within the alternating hydrogenation mechanism, which marks the onset of the NRR process, is recognized as the rate-determining step, meaning it determines the entire reaction rate and acts as a bottleneck [34].

Our observations indicate that the Gibbs free energy change for the initial hydrogenation step on pure TiO_2_ remains relatively small, both before and after anion adsorption. The impact of anion adsorption on the TiO_2_ catalyst varies significantly depending on the type of anion. The Gibbs free energy change for the rate-determining step on pure TiO_2_ is 1.81 eV; for polyatomic anions such as NO_3_^−^, CO_3_^2−^, and SO_4_^2−^, the ΔG increases to 2.35 eV, 2.4 eV, and 2.25 eV, respectively, which is notably higher than the 1.81 eV of pure TiO₂. The notable impact may be ascribed to the intricate molecular compositions of these substances, which cause substantial deformation of the crystal structure and reorganization of the electrical configuration on the surface of TiO_2_. In contrast, the impact of monoatomic anions is relatively moderate. For instance, the ΔG after I^−^ adsorption is 2.16 eV, the highest among monoatomic anions; Br^−^ shows a ΔG of 2.02 eV, Cl^−^ is 1.94 eV, and F^−^ also exhibits a ΔG of 2.02 eV. Despite F^−^ having a stronger electron-withdrawing capability that could potentially lead to lattice distortion and changes in electronic structure similar to polyatomic anions, the larger atomic radii of Cl^−^, Br^−^, and I^−^ result in greater energy barriers. As for I^−^, although it has the largest atomic radius and the lowest electronegativity among the halide anions, its larger size likely causes more severe lattice distortion, significantly raising the energy barrier in the hydrogenation step of NRR, thereby impacting the efficiency.

In a typical photocatalytic process, the photo-induced electrons (e^−^) can be transferred to the conduction band (CB) from the valence band (VB), and the holes ($h^{+}$) can be formed in the VB under visible light irradiation (Equation (1)). Subsequently, the photo-induced e^−^ would react with N_2_ to form NH_3_ directly (Equation (2)), and h^+^ in VB will react with H_2_O molecules to generate O_2_ during the reaction (Equation (3)).

(1) $${TiO}_{2}\overset{h\nu }{\to }{TiO}_{2}(e^{-},h^{+})\#$$

(2) $$N_{2}+6e^{-}+6H^{+}\to 2{NH}_{3}\#$$

(3) $$\begin{array}{c} 3H_{2}O+6h^{+}\to \frac{3}{2}O_{2}+6H^{+} \end{array}$$

(4) $$\begin{array}{c} N_{2}+3H_{2}O\overset{h\nu }{\to }2{NH}_{3}+\frac{3}{2}O_{2} \end{array}$$

Aside from this rate-controlling step, the remaining hydrogenation processes proceed along a spontaneous downhill pathway [35]. Overall, the adsorption of various anions on TiO_2_ hinders the effective activation of the N≡N bond, thereby impeding the initiation of NRR to varying degrees. The effects of polyatomic anions are generally more pronounced, while the influence of monoatomic anions, although milder, still increases the energy barrier and weakens the catalytic activity of TiO_2_.

The individual steps of the alternating mechanism can be expressed as the following reaction equations:

(5) $$\begin{array}{c} N_{2}+*\to *\;N-N \end{array}$$

(6) $$\begin{array}{c} {}*\;N-N+H\to *\;N-NH \end{array}$$

(7) $$\begin{array}{c} {}*\;N-NH+H\to *\;NH-NH \end{array}$$

(8) $$\begin{array}{c} {}*\;NH-NH+H\to *\;NH-NH_{2} \end{array}$$

(9) $$\begin{array}{c} {}*\;NH-NH_{2}+H\to *\;NH_{2}-NH_{2} \end{array}$$

(10) $$\begin{array}{c} {}*\;NH_{2}-NH_{2}+H\to *\;NH_{2}+NH_{3} \end{array}$$

(11) $$\begin{array}{c} {}*\;NH_{2}+H\to *\;NH_{3} \end{array}$$

(12) $$\begin{array}{c} {}*\;NH_{3}\to *\;+NH_{3} \end{array}$$

The corresponding changes in Gibbs free energy (ΔG) can be calculated as follows:

(13) $$\begin{array}{c} △G_{1}=G\left(*\;N-N\right)-G\left(*\right)-G\left(N_{2}\right) \end{array}$$

(14) $$\begin{array}{c} △G_{2}=G\left(*\;N-NH\right)-G\left(*\;N-N\right)-G\left(H\right) \end{array}$$

(15) $$\begin{array}{c} △G_{3}=G\left(*\;NH-NH\right)-G\left(*\;N-NH\right)-G\left(H\right) \end{array}$$

(16) $$\begin{array}{c} △G_{4}=G\left(*\;NH-NH_{2}\right)-G\left(*\;NH-NH\right)-G\left(H\right) \end{array}$$

(17) $$\begin{array}{c} △G_{5}=G\left(*\;NH_{2}-NH_{2}\right)-G\left(*\;NH-NH_{2}\right)-G\left(H\right) \end{array}$$

(18) $$\begin{array}{c} △G_{6}=G\left(*\;NH_{2}\right)+G(NH_{3})-G\left(*\;NH_{2}-NH_{2}\right)-G\left(H\right) \end{array}$$

(19) $$\begin{array}{c} △G_{7}=G\left(*\;NH_{3}\right)-G\left(*\;NH_{2}\right)-G\left(H\right) \end{array}$$

(20) $$\begin{array}{c} △G_{8}=G\left(*\right)+G(NH_{3})-G\left(*\;NH_{3}\right) \end{array}$$

## 3. Computational Methods and Models

To study the changes in the electrical and optical properties of TiO_2_ after the adsorption of anions, we employ the Vienna ab initio simulation package (VASP) to perform first-principles calculations based on density functional theory (DFT). The projected augmented wave (PAW) method employs the Perdew–Burke–Ernzerhof (PBE) generalized gradient approximation (GGA) to characterize the exchange–correlation interactions and core electrons [36]. To solve the problem of underestimation of the band gap that is observed if standard DFT methods are used, the HSE06 hybrid functional was used [37]. By including this, it allowed for a thorough analysis of the electrical characteristics, leading to a more precise representation of the band structure. To avoid spurious interactions arising from the periodic boundary conditions used in the simulations, a vacuum layer of 20 Å thickness was inserted [38]. A plane-wave basis set with an energy cutoff of 550 eV was implemented to ensure computational precision [39]. Moreover, the convergence criterion for energy was set at 10^−5^ eV and for force was set at 0.01 eV/Å so as to allow full relaxation of all geometric structures [40].

The research calculates the adsorption energies using the following formula:

Eads=Etotal−(Esubstrate+Eabsorbate)

The overall energy of the system with the adsorbed species is denoted as Etotal, the energy of the clean substrate is denoted as Esubstrate, and the energy of the isolated adsorbate molecule is denoted as Eabsorbate.

The calculation of the Gibbs free energy change (ΔG) for the stages of the NRR is performed using the following formula:

∆G=ΔE+ΔZPE−TΔS

The reaction energy derived from DFT calculations is denoted as ΔE, the entropy contribution at 298.15 K is denoted as TΔS, and the zero-point energy difference is denoted as ΔZPE [41].

This work utilized a highly refined model of the rutile TiO_2_ (001) surface, which was particularly developed to examine surface interactions and reactions. The lattice constants of the pristine rutile TiO_2_, as reported in the literature, and the relative errors from GGA calculations are summarized in Table 3 [42], demonstrating excellent agreement. The discrepancies in the a and b lattice constants are nearly 0%, while the difference along the c-axis is only 1.26%. The model was expanded to include four distinct atomic layers, comprising a total of 96 atoms, with 32 titanium (Ti) atoms and 64 oxygen (O) atoms.

## 4. Conclusions

This work utilized density functional theory (DFT) to examine the influence of anion adsorption on the electrical and optical characteristics of the rutile TiO_2_ (001) surface. From the results of the present work, it can be concluded that typical anions inherent in water influence the photocatalytic activity of TiO_2_. Indeed, the presence of anions modifies the energy gap between the valence and conduction bands, the distribution of energy levels, and the ability of light absorption. These changes directly influence the ability to inhibit charge carrier separation and recombination. Additionally, complex anions such as NO_3_^−^, CO_3_^2−^, and SO_4_^2−^ significantly increase the energy barrier for the NRR process, thereby reducing the photocatalytic activity of TiO_2_. These findings would help build and refer to a significant theoretical framework and guide to work with TiO_2_ photocatalysts in multicomponent aqueous systems.

## Figures and Tables

**Table 1:** Comparison of Ti-O bond length and E_ads_ before and after anion adsorption for rutile TiO_2_ (001).

Configurations	Average Ti-O Bond Length (Å)	E_ads_ (eV)
TiO_2_ (001)	1.936	/
TiO_2_-Ti-F^−^	1.950	−3.505
TiO_2_-Ti-Cl^−^	1.948	−0.913
TiO_2_-Ti-Br^−^	1.946	−1.075
TiO_2_-Ti-I^−^	1.944	−1.504
TiO_2_-O-F^−^	1.942	−1.545
TiO_2_-O-Cl^−^	1.940	−0.812
TiO_2_-O-Br^−^	1.938	−0.631
TiO_2_-O-I^−^	1.937	−0.603
TiO_2_-NO_3_^−^	1.945	−0.896
TiO_2_-CO_3_^2−^	1.947	−1.278
TiO_2_-SO_4_^2−^	1.950	−3.025

**Table 2:** The Mulliken charges before and after adsorption.

Configurations	Mulliken Charge (e)
TiO_2_ (001)	2.49
TiO_2_-Ti-F^−^	2.28
TiO_2_-Ti-Cl^−^	2.32
TiO_2_-Ti-Br^−^	2.36
TiO_2_-Ti-I^−^	2.24
TiO_2_-NO_3_^−^	2.30
TiO_2_-CO_3_^2−^	2.34
TiO_2_-SO_4_^2−^	2.37

**Table 3:** Comparison of the theoretical model and experimental measurements of the lattice constants for rutile TiO_2_.

Lattice Constants (Å)		
Category	a/b	c
This work	3.78	9.62
Experimental	3.78	9.50
Difference	0%	1.26%

## Data Availability

The original contributions presented in the study are included in the article material, further inquiries can be directed to the corresponding authors.
